# Renal cell carcinoma metastasis to the ovary: a case report

**DOI:** 10.4076/1757-1626-2-7472

**Published:** 2009-07-14

**Authors:** Lawrence Toquero, Omar M Aboumarzouk, Zahir Abbasi

**Affiliations:** 1 Surgical Department, Chesterfield Royal HospitalChesterfield Road, Chesterfield, S44 5BLUK; 2 Burns and Plastics Surgery Department, Morriston HospitalMorriston, Swansea, Wales, SA6 6NLUK; 3 Consultant Urologist Rotherham District General, Moorgate Road, Hospital, Rotherham, S60 2UDUK

## Abstract

A 54-year-old woman referred to a specialist unit for weight loss, lethargy, and a palpable pelvic mass. Thought to have ovarian cancer metastasized to the kidney, underwent a left nephrectomy and para-aortic clearance, with a total abdominal hysterectomy and bilateral salpingo-oophorectomy with peritoneal biopsies. Histology proved it was actually a renal cell carcinoma metastasized to the ovaries. During further follow ups she had developed bone and pulmonary metastasis and died shortly after the diagnosis of metastasis. With only 14 reported cases in the literature increased awareness would aid management of similar cases.

## Introduction

Renal cell carcinoma, also called renal adenocarcinoma or hypernephroma, is a common malignant solid tumour which accounts for 75% of renal neoplasm and 3% of all adult malignancies [1,2]. About 20-30% of patients have distant metastasis during their first presentation, and 50% develop metastasis during follow up [2,3]. RCC, has a 2:1 male to female ration with a mean age at presentation between 50-70 years [2].

## Case presentation

A 54-year-old Caucasian woman was referred to the gynaecology outpatient department by her GP for a 3 month history of lethargy, a 4 stone weight loss, and on examination a non-tender left pelvic mass was palpated. An ultrasound revealed a solid 4 cm mass in the left adnexa. Incidentally, the ultrasound also revealed an enlarged left kidney, with a solid mass occupying the middle and upper poles.

**Figure 1.**
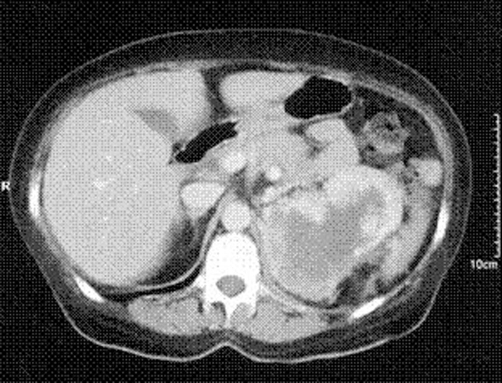
Contrast CT scan of the Left Renal Cell Carcinoma revealing a 10 × 8 cm intensely enhancing heterogeneous mass arising from the upper pole.

**Figure 2.**
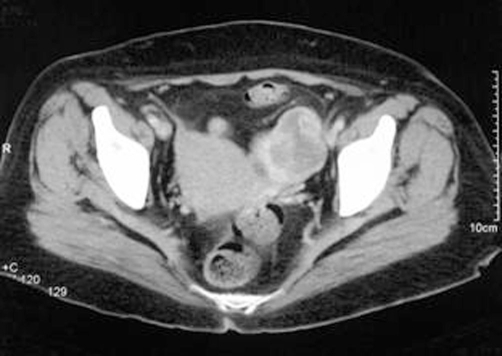
Contrast CT scan of the Left Ovarian metastasis; showing a 4 cm heterogeneous mass with fairly intense enhancement.

A CT scan revealed a 10 × 8 cm intensely enhancing heterogeneous mass arising from the upper pole of the left kidney, consistent with renal cell diagnosis (Figure 1). There was spread to the left para-aortic region at the level of the hilum, where a 1.5 cm lymph node was found. Also the left adnexal region, close to the left lateral margin of the uterus, revealed a 4 cm heterogeneous mass with fairly intense enhancement, which was initially thought to be a pedunculated fibroid (Figure 2).

Thought to be an ovarian tumour, which metastasised to the kidney a joint gynaecological and urological operation was undertaken where she underwent a left nephrectomy & para-aortic clearance, with a total abdominal hysterectomy and bilateral salpingooophorectomy with peritoneal biopsies. Bone scan and chest CT showed no evidence of metastasis anywhere else in her body.

**Figure 3.**
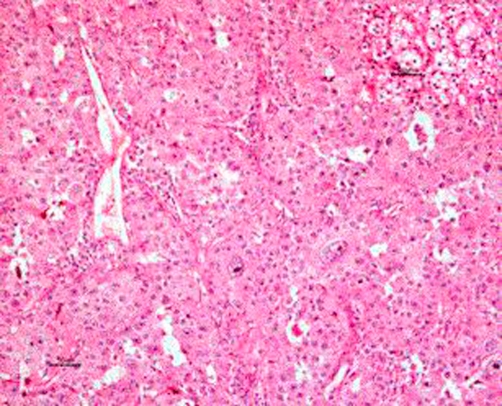
Haematoxylin & Eosin stained section of kidney tumour. Some parts are low grade clear cell (inset top right), but most (main photo) is high grade with eosinophilic cytoplasm, as were the metastases. Scale is 50 mu; low grade and high grade transformation with sarcomatoid features.

**Figure 4.**
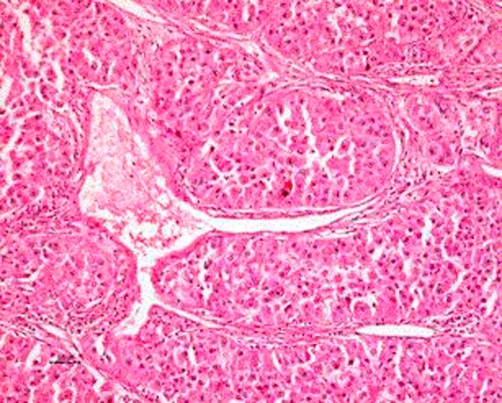
Haematoxylin & Eosin stained section from ovarian metastasis. Same pattern as high grade parts of kidney. Note typical thin walled staghorn vessels, the large one containing strands of fibrin; entirely consistent with metastatic high grade renal cell carcinoma.

The histology report of the kidney was consistent with renal cell carcinoma of low grade and high grade transformation with sarcomatoid features (Figure 3). The grade was a grade 4 of Fuhrman’s Classification system for nuclear grading. The report of the lymph node showed a sarcomatoid high grade metastatic renal cell carcinoma. The left ovary was entirely consistent with metastatic high grade renal cell carcinoma (Figure 4).

She initially improved but 6 months after her operation during a routine follow ups she complained of left shoulder pain and further investigation found lytic lesions in her left proximal humerus and multiple pulmonary metastases. MDT decision was for further palliative radiotherapy and therapy with zoledronate for symptomatic relief. Unfortunately she died 3 months later.

## Discussion

Renal cell carcinoma most frequently metastasize via lymphatic and venous routes to the lungs (50-60%), lymph nodes (36%), bones (30-40%), liver (30-40%), and brain (5%) [3]. RCC is known to metastasis to other sites but these are rare occurrences.

The ovaries are a common site for intraabdominal metastasis and about 6% of ovarian cancers found at laparotomy are secondaries from other sites, commonly stomach, colon, breast, and lymphoma [4]. However ovarian metastasis from renal cell carcinoma is rare. This may be due to the fact that RCC predominates in males; also the mean age group in which RCC presents are of postmenopausal women where the ovaries have undergone vascular sclerosis [5]. More over some metastatic lesions are mistaken for primary ovarian tumours [5].

In one autopsy study, ovarian metastasis was found in 0.5% of cases of RCC [6]. Metastasis to ovaries is thought to occur by retrograde venous embolisation through the renal vein to the ovarian vessels [5,6]. Metastasis through this mechanism exploits the unique anatomy of the left renal and ovarian veins. It mandates incompetent gonadal veins to allow for retrograde venous flow. In fact, two thirds of reported cases arose from a left sided lesion. Thus, it appears that the hallmark for the renal-ovarian axis is its unique venous anatomy.

Only 14 such cases are reported in literature. Out of these, 13 cases were metastasis of RCC of clear cell type and 1 was from a renal pelvis transitional cell carcinoma. Five of these cases were diagnosed as primary ovarian clear cell cancer, whilst renal primary was diagnosed only after proper investigations.

Histological differentiation between clear RCC and Ovarian Clear cell cancer may be difficult. However, certain histological and ultra structural features may help to establish the correct diagnosis. The presence of clear cells with some areas showing a tubulocystic pattern with the characteristic “hob-nail” appearance of the lining epithelium and extra cellular mucin are more typical of clear cell carcinoma of the ovary [7-9]. While clear RCC are round to ovoid and circumscribed by a psuedocapsule of compressed parenchyma and fibrous tissue, rather than a true capsule, they are also hypervascular [10]. The cells also contain numerous lipid vacuoles and long slender microvilli and lack rough endoplasmic reticulum [7-9].

In the ovary the differential diagnosis of clear cell neoplasm includes primary clear cell carcinoma, steroid cell tumour, and dysgerminoma. While metastatic RCC may mimic these entities. Rarely Lipoid cell tumours, Hilus cell and Leydig cell tumours may also be mistaken for metastatic RCC.

However, careful gross and microscopic examination combined with immunohistochemical analyses can aid in the distinction of this metastatic lesion. Tumour markers are another way of differentiating between renal and ovarian clear cell carcinomas. Monoclonal antibodies and are expressed in 80-90% of renal cell tumours but its specificity as a diagnostic marker is still not established. Cytokeratin 7 is expressed in most ovarian cell carcinomas while negative in RCC [8]. CA125 is another marker expressed by epithelial ovarian tumours including clear cell carcinomas but also negative in RCC [8].

Recent studies have shown that systematic therapy can improve survival rates in patients with recurrent metastatic RCC [11]. An increased understanding of RCC molecular biology has lead to the production of vascular endothelial growth factor (VEGF) and mammalian target of rapamycin (mTOR) inhibiting drugs, which inhibit those biological pathways which the tumour cells rely on for growth [11]. VEGF inhibting drugs such as sunitinib, sorafenib, and bevacizumab, and mTOR inhibiting drugs like temsirolimus, have shown some positive results in the increased progression free survival rates [11]. Recent advancements in the treatment of metastatic RCC give numerous management options each with risks and benefits, but with limitations in the data available [11]. There are ongoing studies investigating the risks and benefits of combination of these treatments with the over all aim of maximizing the overall therapeutic benefit and delay the lethal burden of the disease, while improving the quality of life of the patient [11].

After a thorough review of Medline, Pubmed, and the internet, our case represents only the fifteenth reported case of renal cell carcinoma metastasised to the ovary. In addition, it is unique in its presentation in which symptoms of weight loss, lethargy, and loss of appetite started only 3 months prior to her referral.

## Conclusions

Women presenting with an ovarian malignancy may be misdiagnosed. Full abdominal imaging should be performed before exploration. In this review, a delay in diagnosis of the primary lesion from the kidney occurred in over one third of cases, with the presenting symptoms corresponding to the metastatic site rather than the primary lesion. Limited evidence suggest that surgical extirpation of both lesions may lead to long term disease free survival.

Although rare, possibility of ovarian metastasis from renal cell carcinoma should be considered in the differential diagnosis. It is important to identify the primary site by careful histological and histochemical analysis as the course of therapy varies in addition to the prognostic implications. Thus, timely diagnosis of the primary lesions must be pursued.

## Abbreviations

Ca: Cancer
CT: Computed tomography
GP: General practioner
RCC: Renal cell carcinoma
